# A Consumer Segmentation Study of Nutrition Information Seeking and Its Relation to Food Consumption in Beijing, China

**DOI:** 10.3390/foods11030453

**Published:** 2022-02-03

**Authors:** Yin Wang, Jiayou Wang, Qiong Shen

**Affiliations:** 1 Business School, Zhengzhou University, Zhengzhou 450000, China; hnwangyin@zzu.edu.cn; 2 Institute of Agricultural Economics and Information, Guangdong Academy of Agricultural Sciences, Guangzhou 510640, China; jiayou1214@163.com

**Keywords:** nutrition information, consumer segmentation, food consumption, urban China

## Abstract

The aim of this study is to identify consumer groups based on nutrition information-seeking behavior and how it relates to food consumption. Although the Chinese public can now access nutrition information through different channels, research on the segmentation of homogeneous consumer groups seeking nutrition information is lacking. This study closes this research gap and, in doing so, also shows how information seeking is related to dietary behavior. A questionnaire was sent out to a stratified random sample in Beijing, resulting in 448 responses. A cluster analysis using hierarchical methods was conducted, identifying four distinct consumer groups: Multi-Channel (27.43%), Mass Media (20.57%), Moderate (27.88%), and Uninterested (24.12%). The four segments differed significantly concerning food consumption frequencies, food literacy, and sociodemographic characteristics. Consumers who were more involved in nutrition information tended to eat healthier. Our findings indicate that nutrition information is worth promoting, but this kind of intervention is not a cure-all. Targeted interventions should focus on uninterested populations by providing non-informational nudging strategies to promote healthy eating behaviors. This study contributes to the identification of meaningful profiles for targeted interventions, particularly as regards uninterested or unreached consumers.

## 1. Introduction

A poor diet is associated with an increased risk of cardiovascular disease, diabetes, and several cancers [1,2]. China is undergoing a remarkably rapid, but undesirable, shift toward a stage in nutrition transition that is dominated by a high intake of meat products as well as fast food and sugar-rich soft drinks [3,4]. According to the Chinese Residents Nutrition and Chronic Disease Status Report (2020), the problem of obesity and diet-related diseases among Chinese residents has become increasingly prominent. Providing intuitive and straightforward nutrition information is increasingly perceived as an essential tool for combatting unhealthy food choices and for improving public health [5,6]. Particularly during the COVID-19 pandemic, the provision of nutrition information is an important intervention to address the nutritional insufficiencies caused by the lack of supply [7].

In China, nutrition labeling is compulsory for prepackaged foods [8]. The Chinese government has begun to actively promote the Chinese Dietary Guidelines (CDG), which suggest different food groups based on the different energy levels required [9,10]. Additional sources of nutrition-related information are available to help consumers make healthy food choices. At present, mass media, especially new media, has become the primary channel for the dissemination of nutrition information [11]. However, little is known about the status of consumers seeking various sources of nutrition information, what motivates consumers to seek nutrition information, and the different types of food consumption related to nutrition information usage. This paper aims to shed light on these issues.

Consumers do not respond to various sources of nutrition information in the same way, and the one-size-fits-all approach to health campaigns or interventions has ignored the needs of different segments in terms of behavioral change strategies [12]. Consumer segmentation helps us to better understand the attitudes and behaviors of specific consumer groups rather than learning how an “average” consumer thinks and behaves [13]. One representative study found that consumers can be segmented based on their use of and attitudes toward nutrition labels, and that such segmentation can provide valuable information for a targeted communication approach [14]. A few recent studies have identified consumer groups based on the use of and trust in information sources [15] and profiled the segments using sociodemographic variables [16], purchase motives [17], and consumption behavior [18]. To the best of our knowledge, our study is the first in which consumer segments based on nutrition information-seeking behaviors have been related to food consumption in China.

To provide meaningful profiles of different groups, variable selection should be based on theory. Previous studies about the determinants of nutrition information seeking can be categorized into two parts: The first is the factors affecting perceived benefit. If consumers perceive the essential link between their current diet and future health, they may be more conscious and motivated to seek nutrition information [19]. Higher interest in healthy eating has been associated with more information usage and benefit [20]. A higher level of food involvement seems to be associated with higher motivation [21]. The second is the factors affecting the cost of searching. Information-searching costs are mainly impacted by opportunity cost and efficiency [22]. Empirically, employment situation and income are included in the analysis to help understand the effects of time pressure [23,24]. More educated consumers are more likely to use nutritional labels [25]. Likewise, knowledge of nutrition can facilitate the search for nutrition information by increasing its perceived benefits and increasing the effectiveness of its use, thus reducing the cost [26]. It is also essential to take sociodemographic characteristics into account [27,28,29]. 

It would also be helpful to determine if there are differences between consumer groups that use or do not use nutrition information in their food choices. Nutrition labeling not only provides information on the safety of a product but also reminds consumers how it is important to choose healthy products [30]. When exploring the role of leading factors influencing food quality from 1994 to 2010 in the US, the results showed that the most significant contributor is the increased use of nutrition facts tables and health claims [31]. In agreement with this finding, several studies have revealed links between the use of nutrition labels and healthy food choices [32,33,34]. However, other empirical research studies have shown that providing nutrition labels on foods does not effectively improve the health of consumer food choices [35,36]. 

As shown in the literature review, various factors and complex effects across nations mean that it is necessary to examine this issue in the biggest developing country. The purpose of this research is to explore the nutrition information-seeking behavior using segmentation based on various information sources and to then profile the segments using seeking motives, sociodemographic variables, food attributes, and consumption behavior. The main contribution of this paper lies not only in the study of nutrition information-seeking behavior in a new geographic context but also in the identification of meaningful profiles for targeted interventions, particularly in regard to uninterested or unreached consumers. The rest of the paper is organized as follows: Section 2 discusses the materials and the method, while Section 3 presents the results. The Discussion section presents the theoretical as well as practical implications of the findings along with the limitations of the study and future research directions. Section 5 briefly concludes the paper.

## 2. Materials and Methods

### 2.1. Data Collection

The survey was conducted in Beijing, the capital of China. The research data were obtained from 500 face-to-face interviews, which were conducted in January 2017 with consumers from the five major urban areas of Beijing, Haidian, Dongcheng, Xicheng, Fengtai, and Chaoyang. The questionnaire underwent pilot tests with a small sample of consumers to ensure question clarity and to avoid interview mistakes (*n* = 25). There were 448 usable samples in this study, as complete responses to crucial information and logical consistency were necessary for the statistical analysis conducted in the research.

Sample selection was conducted by stratified random sampling with proportional allocation to gender and age to avoid the under or over representation of the consumer profiles according to the sociodemographic statistical data from the Beijing Statistical Yearbook 2017. The questionnaires were administered by specially trained students. To achieve the maximum possible variation, the survey was conducted on different days and at different times and locations. The interviewers approached the respondents at supermarkets, shopping centers, or public parks in the main urban areas. Past literature suggested that consumers in these locations represent different levels of consumption, allowing us to avoid biased sample selection [37]. The selection criteria were adults aged above 18 years of age who had been living in Beijing for more than one year (relatively stable living condition). Before beginning the survey, the specific goals of the study as well as the study procedures and average time required for participations were explained to the participants. The participants were also informed that they could end their participation at any time and that their involvement would not require any effort other than filling in the questionnaire. No financial compensation was provided to the participants.

The final sample included 52.1% female participants and 47.9% male participants. The participants included different age groups: a young age group (<40 years old), which accounted for 46.1% of the participants, a middle age group (40–60 years old), which accounted for 35.9% of the participants, and an old age group (>60 years old), which accounted for 18% of the participants. Approximately half of the sample had received a tertiary level of education. Colleges and universities are widely distributed in Beijing, so there were more respondents with bachelor’s degree or above in the sample. The sample structure was similar to the census data of the resident population in Beijing (Table A1).

### 2.2. Questionnaire Design

The research team developed a self-administered questionnaire containing questions regarding nutrition information-seeking behavior, food literacy, attitudes to food attributes, food consumption frequency, and sociodemographic details. In order to investigate the validity of the questionnaire, we tested the content validity and face validity [38]. To determine the content validity, the test was assessed by five experts with a nutrition background. We asked the experts to score each item out of 5 based on the level of appropriateness of the item. The CVI of each item was above 4. In terms of face validity, the questionnaire underwent pilot tests with a small sample of consumers (*n* = 25). All of the respondents were interviewed after completing the questionnaire. The majority found that the layout of the questionnaire was clear and that the font size and length of the questionnaire were accurate. The questionnaire’s instructions were considered easy to understand by 80% of the respondents. The specific contents of the questionnaire were as follows:

Nutrition information-seeking behavior: Participants were asked to assess how frequently they used nutrition information, with responses ranging from never to very often, on a five-point scale. Specifically, seven information sources were included: the internet, TV programs, newspapers and radios, government campaigns, dietitians, friends and relatives, and food labels. The internal reliability of the nutrition information-seeking scale was good, with Cronbach’s α = 0.687, *n* = 7.

Food literacy: Food literacy, the capacity to gather, understand, process, and use relevant information to navigate the food system, is a critical concept that inspires food policies worldwide [39]. At the individual level, food literacy is considered an important variable that can determine one’s ability to assess and meet individual nutrition needs [40]. To evaluate the food literacy of consumers, we adopted a self-reporting approach that allowed participants to describe their eating habits and food skills, including their (1) interest in healthy eating, (2) nutrition knowledge, and (3) diet-health consciousness.

- Interest in healthy eating: Three statements were used to evaluate the participants’ interest in healthy eating using a 5-point scale ranging from disagreement to absolute agreement. The first statement was “I believe that healthy eating is important”, the second was “I expect to know a lot about nutrition information and recommendations on a healthy diet”, and the third was “I think that if I am properly informed about the nutrition information, I could apply it to my diet” [20]. The internal reliability of the interest in healthy eating scale is also excellent, with Cronbach’s α = 0.865, *n* = 3.
- Nutrition knowledge: Nutrition knowledge can take two general forms: knowledge of principles and knowledge of a food’s specific nutrient content [41]. Knowledge of principles was measured using two components drawn from the Chinese Dietary Guidelines. The first component measured the participants’ awareness of a dietitian’s recommendations about vegetables and fruits, grains, fish/poultry, milk and soybean products, oil, fried food, and salty food. For these seven items, participants stated whether or not the recommendations told them to have more or less of the food. The second component contained seven statements about dietary habits and their health implications. Such statements about healthy eating patterns were generally poorly understood by the Chinese public. We asked participants to determine if they agreed or disagreed with these statements. We assumed that people with better knowledge of nutrition would be more likely to provide the right answers. For this portion of the questionnaire, correct answers received a score of one, and wrong answers received a score of 0. The total possible score for this section was 14. Specific nutrition knowledge was measured by six questions to determine how much the consumers knew about protein, cholesterol, Vitamin C, fats, and salt. Participants were asked to choose the main sources of high-quality protein and Vitamin C and to determine which food had the most cholesterol or fat. Since the excessive use of salt is an important dietary problem in China [42], we also assessed the consumers’ knowledge of the maximum amount of salt in their diets. The maximum possible score for this section was 7. As such, the total possible score for all of the questions related to nutrition knowledge was 21. Cronbach’s α = 0.511, *n* = 20.
- Diet-health consciousness: To measure general diet-health consciousness, one statement was used to evaluate the participants’ awareness of a healthy diet using a 5-point scale ranging from disagreement to absolute agreement with the following statement: “I think food and nutrition play an important role in maintaining health”. To measure specific diet-health consciousness, four unhealthy eating habits were included, such as excessive cholesterol intake, excessive sugar intake, excessive fat intake, and not consuming enough dietary fiber. Participants were asked to judge whether or not these habits might lead to health problems. Correct answers were given a score of 1, and wrong answers or answers where the participant stated that they did not know were given a score of 0. A total score of 9 was possible for this section. Cronbach’s α = 0.512, *n* = 4.

Attitudes toward food attributes: Consumer food choice behaviors are sophisticated processes, since individuals have to trade off various food attributes and make decisions. The participants had to respond whether they value four food attributes: nutrition, price, taste, and convenience, according to their consideration when making decisions. Cronbach’s α = 0.823, *n* = 4.

Food consumption frequency: One of the aims of our study was to investigate how nutrition information and awareness affect food behaviors. Given this purpose, we chose to add food frequency questions to measure the participants’ stated food consumption behaviors. We identified five food groups: vegetables, fruits, fish/seafood and poultry, milk and dairy products, and fast food. Food intake was evaluated on a 6-point scale corresponding to the following choices: more than once daily, once daily, 4–6 times weekly, 1–3 times weekly, 1–3 times monthly, and seldom or never. To facilitate the statistical calculations, we coded the responses according to the following rules: more than once daily was coded as 14 times weekly (assumed twice daily); once daily was coded as seven times weekly; 4–6 times weekly was coded as five times per week; 1–3 times weekly was coded as two times per week; 1–3 times monthly was coded as 0.5 times weekly, and seldom or never were coded as 0 times weekly. Cronbach’s α = 0.608, *n* = 5. In Chinese eating habits, vegetables and fruits are daily foods. The difference in frequency would not be too large, and therefore, it was necessary to better understand the portions for each meal. For vegetables, the participants had to choose quantities ranging from less than one Liang (a Chinese traditional unit of weight, 1 Liang = 50 g) (0.5 portions), one to two Liang (1 portion), three to four Liang (2 portions), five to six Liang (3 portions), and more than six Liang (4 portions). For fruits, we used an apple (approximately 200–250 g) for participants to refer to, with less than 200 g being equal to 0.5 portions; 200–300 g being equal to 1 portion; 400–500 g being equal to 2 portions; and more than 600 g being equal to 3 portions. The portion multiplied by the food consumption frequency resulted in the total consumption of the two items. Cronbach’s α = 0.406, *n* = 2. In addition, the participants were asked to consider if their current diet was healthier than it was three years ago, and this statement was rated on a 5-point scale from disagreement to total agreement.

Social–demographic characteristics: The survey ended with questions regarding the participant’s gender, age, educational attainment, monthly household income, family size, and other household characteristics. The participants were also asked whether they had a nutrition-related background and how often they purchased food. Additionally, we asked whether the participants followed a special diet, such as a low-fat, low-sugar, or low-salt diet.

### 2.3. Data Analysis

Cluster analysis was based on the self-reported frequency of how often the participants sought nutrition information. More specifically, we performed a hierarchical cluster analysis using Ward’s method and square Euclidean distance as a distance measurement. In the case of uncertain classification, we made a tentative choice of 3–6 to determine the proper cluster. Following Demirmen’s criteria for selecting groups by considering the most meaningful partition subjectively in terms of the research aims, we found four segments where the distance between the group gravity centers was relatively far. Finally, findings from the multivariate analysis of variance (MANOVA) procedures demonstrated that the means of the seven different information sources were significantly different among the four chosen clusters (*p* < 0.01). In addition, based on the hypothesis of equal covariance matrices, Duncan’s multiple range tests confirmed that each group was significantly different from the others. Thus, the four-segment solution was proven to be reasonable and robust. After the cluster analysis, we performed additional MANOVA analyses to discover whether and how the identified clusters differed concerning attitudes toward food attributes, food consumption frequency, and the main determinants of nutrition information-seeking behavior. These analyses also helped to identify the characteristics of the individuals who fell into each of the four segments. We conducted three general empirical tests and investigations: (1) We performed association tests to determine whether cluster membership could be related to attitudes toward food attributes and food consumption frequency. (2) We investigated and tested how the various determinants of nutrition information-seeking behavior, such as nutrition knowledge, diet-health consciousness, and interest in healthy eating, differ across segments. (3) Lastly, we investigated and tested how social demographic characteristics, including gender, age, education level, special diet, nutrition background, food-buying frequency, monthly household income, the presence of children, and the presence of elderly residents, all vary across segments. LSD and Dunnett T3 multiple range tests were used on a post hoc basis to determine which clusters differed from each other.

## 3. Results

### 3.1. Characteristics of Nutrition Information-Seeking and Segmentation Analysis

Mass media was the primary channel through which the consumers obtained nutrition information. In Table 1, the frequency at which nutrition information was sought via TV shows was the highest, which was followed by the Internet. Communication with relatives and friends about nutrition information can be a method of peer nutrition education and was the next most common information source. Food labels were also commonly used sources. Government campaigns and consultations with dietitians were not widely used, and more than half of the respondents said that they were “rarely” seeking nutrition information through these channels.

Cluster analysis, which was based on the seven information source variables discussed in the previous section, identified groups who received nutrition information from similar sources. The participants included in segment 1 were characterized by a firm intention to use all available media to obtain nutrition information. Thus, we labeled this segment as “Multi-Channel Seekers” (mean frequency was 3.49). Approximately 27% of the participants were classified in this group. The consumers in segment 2 were more likely to rely on mass media and peer education from family and friends. However, they were less willing to use government campaigns and food labels. This segment was labeled as “Mass-Media Seekers” (mean frequency was 2.94) and contained 20.58% of the consumers. Consumers from segment 3, the largest group representing 27.88% of the participants, were identified as “Moderate Seekers” (mean frequency was 2.71). Compared to the representatives in the “Multi-Channel Seekers” group, the participants grouped into segment 3 are particularly inclined to search for nutrition information through the internet, from websites, and through social media. In addition, the segment 3 consumers had relatively higher involvement with food labels than Mass-Media Seekers did. Segment 4 was called “Uninterested Seekers” (mean frequency was 2.26) and contained approximately 24% of the participants. Taking into account that all of the sources of nutrition information used were below the mean of the sample, the consumers in segment 4 represented the lowest levels of nutrition information-seeking behavior. The descriptive analysis of the mean values of the four segments is presented in Table 1.

### 3.2. Differences in Segments’ Food Literacy

The results revealed differences between the clusters based on the three variables (Table 2). Multi-Channel Seekers were the most interested in healthy eating (M = 13.67), which were followed by Mass-Media Seekers, and Moderate Seekers, while Uninterested Seekers were the least interested in healthy eating (M = 11.85). As for diet-health consciousness, participants in the Multi-Channel, Mass-Media, and Moderate segments had a closed outcome. Moderate Seekers (M = 8.21) reported significantly higher consciousness about their diet and health than Uninterested Seekers did (M = 7.74). However, even though it was determined that the Multi-Channel Seekers had the highest level of nutrition knowledge, few statistical differences were noted across the four segments. 

Table 3 shows the differences between the clusters of consumers in terms of gender, age, education, and other sociodemographic variables. The gender composition across each segment was very similar. The consumers in the Moderate Seekers segment were the youngest (M = 37.43). In terms of age structure, Multi-Channel Seekers were mainly middle-aged consumers, while Mass-Media Seekers comprised both young and elderly participants. Uninterested Seekers were the oldest group. In agreement with previous studies, we found that education levels generally increased from the Uninterested to the Multi-Channel segments, with Moderate Seekers having the highest levels of education (M = 15.64). The Moderate Seeker segment had the highest mean value for household income (M = 2.89), and this value was significantly higher than the mean value of the Multi-Channel Seekers and Uninterested segments. As for other characteristics, the Multi-Channel Seekers stood out. They had the highest levels in terms of participants eating a special diet (M = 52.59), nutrition-related background (M = 10.34), food buying frequency (M = 4.04), and family size (M = 5.56).

### 3.3. Differences in Segments’ Attitudes to Food Attributes and Food Consumption Frequency

The survey asked how participants value general food attributes, such as nutrition, price, taste, and convenience. Attitudes toward these food attributes are subjective and involve a behavioral trade-off. Consumer attitudes toward nutrition were positive, although they varied significantly between the four segments (M = 76.79%). Table 4 shows a comparison of the food attitudes in the four clusters. Specifically, the Multi-Channel Seekers displayed the highest values for nutrition (M = 85.09%), and the Uninterested Seekers showed the lowest values for nutrition (M = 62.24%). Both the Multi-Channel Seekers and Moderate Seekers placed a significantly higher value on nutrition than the Mass-Media Seekers did. A high frequency of nutritional information use was associated with placing importance on healthier products. This result corresponds to the outcome of interest in terms of healthy eating. In addition to nutrition, Mass-Media Seekers valued price and convenience more than the other consumer segments did, while the Moderate Seekers had the highest preference for taste.

Overall, the Multi-Channel and Mass-Media consumers reported eating healthier foods more frequently than the other clusters did. Specifically, the Multi-Channel Seekers, who valued food nutrition the most, exhibited relatively healthy food consumption patterns, as indicated by their relatively high consumption of fruits and fish/poultry, and their low frequency of fast food consumption. This segment also consumed more vegetables and milk/dairy products than the Uninterested group did. The Mass-Media Seekers consumed more portions of vegetables and more milk than the other clusters and had a significantly higher consumption frequency of fruits than the Uninterested segment did. The Moderate Seekers, the youngest group who valued taste the most, had the highest consumption of fast food. However, the participants in this segment consumed even lower frequencies of vegetables and fish than the Uninterested segment did. However, the Moderate Seekers consumed the highest quantity of fruits among the four clusters and had a relatively high milk/dairy product intake. Uninterested consumers reported eating healthier foods less frequently than the other groups, reporting a lower number of servings of fruit and milk than the other groups. Additionally, the Uninterested consumers reported a relatively high intake of unhealthy products such as fast food.

## 4. Discussion

This study is the first to create correlations between consumer segments based on nutrition information and dietary behaviors in China. The primary objective was to identify consumer groups based on their nutrition information-seeking behavior in China. The second objective consisted of comparing sociodemographic characteristics, food literacy, attitudes toward food attributes, and the food behaviors of the groups. Based on the findings of the cluster analysis, four segments were taken into account to describe how the participants used nutrition information from a large set of sources. In addition, significant differences were observed across the different segments in terms of food literacy, food attitudes, and food consumption. Using the survey data, we found that consumers who were more involved with nutritional information, such as Multi-Channel Seekers and Mass-Media Seekers, tended to eat healthier. Their relatively healthy food choices resulted in them reporting a higher intake frequency of vegetables, fish, and milk and a lower intake of fast food than the other segments. Our results indicated that seeking nutrition information is positively related to one’s ability to assess their current food situation. Additionally, since consumer attitudes toward food attributes influence food choices, one of the reasons why the Multi-Channel Seekers demonstrated a higher level of healthy eating behaviors could be due to the emphasis that they place on the nutritional attributes of food.

Traditional healthy-eating promotions generally treat all consumers as being homogenous and with similar needs. Therefore, this type of broad communication approach may be ineffective and costly. Consumer segmentation can help with this targeting process. Our results support a targeted communication strategy that identifies consumer groups to motivate their nutrition information seeking and to improve dietary quality. Multi-Channel and Mass-Media Seekers were more easily reached by nutrition information than the other two groups were. Since these groups were relatively conscious of their diet and health, a communication strategy that is targeted at these groups would not have to convince but instead simply inform. Indeed, half of the representatives from the Multi-Channel group were on a special diet, and they might need more information that provides specific dietary guidance. The higher proportion of nutritional background in the Multi-Channel group partly explained why consumers were willing and able to search for nutrition information. This finding provides a significant implication for public policy, which is to popularize nutrition education when people are students. Food education was introduced in China in 2006. Although there has been no systematic, comprehensive food education system at the national level [43], developed parts of China have taken the lead in implementing food education [44]. The importance of food education not only lies in improving the health of the population and mitigating existing problems in food consumption behaviors but also in creating a social environment that is favorable for the formation of healthy eating preferences in young people, contributing to the long-term prosperity and development of human society. 

The Mass-Media Seekers had a relatively high inclination to seek nutrition information from mass media but a low inclination to read food labels when buying foods. A targeted strategy that could inspire Mass-Media Seekers to have greater access to nutrition information is to promote nutrition labels in mass media. The present research suggested that food labels worked by filling a gap in the knowledge for groups of individuals who already had healthy food preferences and an intent to eat healthily but a lack of information at the point of purchase [32]. There is consistent evidence in the present study that nutritional tables were widely used by the consumers in the Multi-Channel and Moderate clusters with high levels of nutrition knowledge, healthy eating behaviors, and high incomes. There is a low rate of utilization of food nutrition information in China, and ignorance and a belief that information is not useful are the primary cause of this [45]. As such, smart nutrition labeling policies should first aim to maximize consumer response through wide recognition and acceptance. A mass media education program should be implemented to enhance consumer understanding and subjective trust in the use of nutrition labels. Furthermore, public authorities should increase their efforts to monitor the industry so that it adheres to high labeling standards, including the accuracy, completeness, and visibility of the information sought by the target population [46].

In addition, we found two clusters that might be more difficult to reach simply by providing nutrition information because they use few information channels: the Moderate Seekers and the Uninterested. Moderate Seekers, the largest segment in the research, were the youngest and had the highest educational attainment level. Consumers in this group had a heightened awareness of diet and health and were interested in a healthy diet but were unlikely to seek nutritional information. They showed interest in using the internet as well as food labels sometimes. However, improvement for this segment could come from reducing their relatively high consumption of fast food. Thus, a targeted approach could focus on the health benefits of fresh food preparation. Social media can be a quick, inexpensive, and straightforward way for Moderate Seekers to obtain targeted nutrition education. In China, social media platforms such as blogs, WeChat, and we media have become a critical way to obtain nutrition information and to improve health literacy. However, due to the network’s openness, anyone can publish and disseminate information, resulting in uneven information quality and severe information pollution [47]. It is difficult to make a distinction between the authenticity of the information. Therefore, the characteristics of false nutrition information should be effectively identified and then protected or eliminated, with a view to fundamentally improve the quality of the network and purify the information environment. Moreover, on social media, opinion leaders serve as an important bridge between the government and the public. Nutritional information can be effectively communicated to the public by establishing authoritative opinion leaders [48].

Uninterested consumers are the most difficult to reach because they had the lowest nutrition information search frequencies. The participants in this group were older and comprised a greater proportion of males. In addition, the Uninterested consumers had the lowest level of educational attainment and food literacy. Furthermore, the Uninterested segment seemed to value price higher and nutrition lower than the other segments. This group had the lowest consumption frequencies of healthy foods, e.g., fruits and dairy products. The inadequate use of different sources of information in combination with low dietary literacy suggests that attempting to consciously stimulate and educate those not interested in seeking nutrition information would not improve the eating behaviors of this group. Promoting nutrition labels or improving social media may not help consumers who are uninterested, as they would not refer to nutrition information. Alternative approaches based on behavioral economics have recently picked up momentum. These approaches are based on subtle changes in the eating environment. Non-informational strategies such as healthier default options significantly promote healthy food choices without restricting the offer or reducing satisfaction in a variety of locations such as schools, workplace canteens, and restaurants [49,50]. Environmental weight-control cues, e.g., thin, human-like sculptures, drive decisions unconsciously and play a pivotal role in reducing calorie intake [51]. Similarly, it has been demonstrated that offering a variety of vegetables, e.g., carrots, broccoli, and snow peas, increased consumption compared to serving only one of these vegetables on its own [52]. These “nudges” are characterized by relatively significant effects on choice in an altered environment, relatively low costs, and little consumer resistance [53].

The main limitations of the present study are the geographical area of the survey performed and the self-reported methodology. Therefore, the survey, which was conducted in the capital city, is not representative of the general population of China. It is challenging to make generalize the findings. Future studies could extend the survey and make comparisons between urban and rural areas. Although self-reported and subjective opinions provide useful insights into consumer behavior, consumers in China probably suffer from a social desirability bias. People of Eastern cultures are likely to provide more socially desirable responses that are compatible with the predominant cultural dimensions in their home countries [54]. In this respect, the actual frequency of seeking nutrition information by the participants may be less than their stated frequency, which means that the findings have to be carefully interpreted. Although content validity and face validity were adopted, there may still be factors affecting validity. In the future, we need to improve the validity, including the addition of non-self-reported scales. Moreover, considering the increasing frequency of food being available away from home and buying food online, future studies on nutrition information intervention in these fields are recommended.

## 5. Conclusions

To the best of the authors’ knowledge, this study is the first to identify consumer segments on nutrition information-seeking behaviors and its relationship to food consumption in China. These findings are of relevance not only to China but also to other developing and developed countries. The results indicate that consumers who are more involved in nutrition information tend to eat healthier. Therefore, nutrition information is worth promoting, as it is of interest to some consumer groups. However, it must be acknowledged that information intervention is not a cure-all. Uninterested consumers cannot be reached by such information interventions. Therefore, in light of our findings, we propose targeted interventions that drive healthy food choices across these four segments. Intelligent nutritional policies for healthy eating should accordingly prioritize three areas of action. Top priority should be given to policies that create an effective information environment. Policies should ensure that those who wish to obtain nutritional information have the opportunity and capacity to do so. To ensure that nutrition information has a direct impact on consumer food choices, policies should be based on specific consumer clusters and should clearly identify barriers, such as the scarcity of capacity, mobility, or social support. A second priority is to provide a supportive social environment through school food education to shape healthy food preferences among youth. Therefore, the third priority is to modify the food environment with non-informational nudging strategies to promote healthy eating behaviors.

## Figures and Tables

**Table 1:** Descriptive of the segments and MANOVA analysis.

Segments	Multi-Channel Seekers (*n* = 124)		Mass-Media Seekers (*n* = 93)		Moderate Seekers (*n* = 126)		Uninterested Seekers (*n* = 109)		Total Sample (*n* = 452)		F(3448)
	Mean	SD	Mean	SD	Mean	SD	Mean	SD	Mean	SD	
internet	3.47 ^b^	1.108	3.88 ^a^	0.832	3.91 ^a^	1.004	1.96 ^c^	0.838	3.31	1.240	99.30 ***
TV shows	3.89 ^a^	0.876	3.53 ^ab^	1.089	2.74 ^c^	0.965	3.37 ^b^	1.086	3.37	1.087	28.63 ***
newspapers and radios	3.69 ^a^	0.932	3.28 ^b^	0.852	2.14 ^d^	0.846	2.42 ^c^	0.955	2.87	1.102	77.07 ***
government campaign	3.21 ^a^	0.819	2.15 ^b^	0.722	1.76 ^c^	0.650	1.75 ^c^	0.596	2.24	0.934	115.94 ***
dietitians	2.75 ^a^	0.934	1.78 ^b^	0.705	1.63 ^bc^	0.678	1.53 ^c^	0.554	1.94	0.890	69.94 ***
relatives and friends	3.45 ^a^	0.896	3.53 ^a^	0.892	2.98 ^b^	0.925	2.72 ^c^	1.035	3.16	0.991	18.15 ***
food labels	3.99 ^a^	0.924	2.44 ^c^	0.800	3.78 ^b^	0.725	2.06 ^d^	0.831	3.15	1.170	153.54 ***

*** *p* < 0.01 for all F values. When a different letter appears in the segments, it means that they are significantly different at the 5% level; when the same letter appears in the segments, it means that no difference is found between each pair of segments. The same is true below. The mean level of a, b, c, and d shows a decreasing trend.

**Table 2:** Food literacy of the four clusters.

Variables	Multi-Channel Seekers (*n* = 116)	Mass-Media Seekers (*n* = 91)	Moderate Seekers (*n* = 124)	Uninterested Seekers (*n* = 107)	Total Sample (*n* = 438)	F(3434)
	M(SD)/%	M(SD)/%	M(SD)/%	M(SD)/%	M(SD)/%	
Interest in healthy eating	13.67 ^a^ (1.928)	12.54 ^bc^ (1.951)	12.76 ^b^ (2.101)	11.85 ^c^ (2.708)	12.73 (2.284)	13.10 ***
Nutrition knowledge	15.16 (2.560)	14.80 (2.320)	15.09 (2.505)	14.50 (2.889)	14.90 (2.588)	1.54
Diet-health consciousness	8.16 ^ab^ (1.087)	8.09 ^ab^ (1.061)	8.21 ^a^ (0.998)	7.74 ^b^ (1.462)	8.06 (1.173)	3.74 **

** *p* < 0.05, *** *p* < 0.01. The mean level of a, b, and c shows a decreasing trend.

**Table 3:** Sociodemographic profiling of the segments.

Variables	Multi-Channel Seekers (*n* = 116)	Mass-Media Seekers (*n* = 91)	Moderate Seekers (*n* = 124)	Uninterested Seekers (*n* = 107)	Total Sample (*n* = 438)	F(3434)
	M(SD)/%	M(SD)/%	M(SD)/%	M(SD)/%	M(SD)/%	
gender (%)						0.27
male	46.55	48.35	45.97	51.40	47.95	
female	53.45	51.65	54.03	48.60	52.05	
age (years)	44.72 ^b^ (12.136)	44.45 ^b^ (16.121)	37.43 ^c^ (11.116)	54.06 ^a^ (15.912)	44.88 (14.997)	27.96 ***
age (category%)						26.37 **
age 18–30	12.09	25.27	34.68	12.15	21.23	
age 31–40	27.59	25.27	33.06	12.15	24.89	
age 41–50	32.76	17.58	20.97	13.08	21.46	
age 51–60	16.38	8.79	7.26	25.23	14.38	
age_60 above	11.21	23.08	4.03	37.38	18.04	
education (years)	15.18 ^ab^ (2.283)	14.60 ^b^ (2.691)	15.64 ^a^ (2.427)	12.79 ^c^ (3.397)	14.61 (2.918)	23.58 ***
education (category%)						23.00 ***
middle school	6.90	8.79	4.84	29.91	12.33	
high school	9.48	19.78	9.68	23.36	15.07	
technical school	28.45	12.09	13.71	15.89	17.81	
bachelor	38.79	49.45	44.35	23.36	38.81	
graduate	16.38	9.89	27.42	7.48	15.98	
special diet (%)	52.59 ^a^	32.97 ^b^	39.52 ^ab^	36.45 ^ab^	40.87	3.35 **
nutrition-related background (%)	10.34 ^a^	8.79 ^ab^	4.84 ^ab^	1.87 ^b^	6.39	2.71 **
food buying frequency	4.04 (0.879)	3.74 (0.917)	3.79 (0.965)	3.71 (1.108)	3.83 (0.977)	2.76 **
family size	3.56 ^a^ (1.066)	3.09 ^b^ (1.161)	3.39 ^ab^ (1.187)	3.15 ^b^ (1.235)	3.31 (1.174)	3.76 ***
monthly household income (category%)	2.51 ^b^	2.67 ^ab^	2.89 ^a^	2.42 ^b^	2.63	4.20 ***
income under 5000	15.52	8.79	5.65	19.63	12.33	
income 5000–10,000	41.38	42.86	35.48	42.99	40.41	
income 10,001–18,000	25.86	28.57	33.06	20.56	27.17	
income 18,001–30,000	11.21	12.09	16.13	9.35	12.33	
income 30,000 above	6.03	7.69	9.68	7.48	7.76	

** *p* < 0.05, *** *p* < 0.01. The mean level of a, b shows a decreasing trend.

**Table 4:** Attitudes related to food attributes and food consumption of the four clusters.

Variables	Multi-Channel Seekers (*n* = 114)	Mass-Media Seekers (*n* = 88)	Moderate Seekers (*n* = 118)	Uninterested Seekers (*n* = 98)	Total Sample (*n* = 418)	F(3414)
	M(SD)/%	M(SD)/%	M(SD)/%	M(SD)/%	M(SD)/%	
Attitudes to food attributes (%)						
nutrition	85.09 ^a^	75.00 ^ab^	82.20 ^a^	62.24 ^c^	76.79	6.26 ***
price	36.84 ^ab^	53.41 ^a^	34.75 ^b^	44.90 ^ab^	41.63	2.98 **
taste	34.21	38.64	47.46	45.92	41.63	1.77
convenience	5.26	14.77	7.63	12.24	9.57	2.19 *
eating healthier	4.31(0.863) ^a^	3.90(0.910) ^bc^	3.98(0.762) ^b^	3.59(1.044) ^c^	3.96(0.926)	11.47 ***
Food consumption frequency						
vegetable	30.59(12.852) ^ab^	32.85(12.484) ^a^	29.10(13.879) ^b^	30.63(13.137) ^ab^	30.66(13.159)	1.37
fruit	11.63(9.596) ^b^	11.60(8.848) ^ab^	13.19(10.493) ^a^	8.27(6.340) ^ac^	11.28(9.205)	5.44 ***
fish/poultry	2.40(2.676)	3.71(15.006)	1.91(1.973)	2.01(1.905)	2.45(7.164)	1.26
milk/dairy product	5.26 (3.935) ^ab^	6.01 (3.740) ^a^	5.49 (3.781) ^ab^	4.47 (3.662) ^b^	5.36(3.799)	1.82
fast food	0.24(0.499)	0.40(1.583)	0.50(1.028)	0.42(1.164)	0.39(1.101)	1.08

* *p* < 0.1, ** *p* < 0.05, *** *p* < 0.01. The mean level of a, b, and c shows a decreasing trend.

## Data Availability

The data presented in this study are available on request from the corresponding author.

## Appendix A

**Table A1:** A comparison of the sample with the resident population in Beijing (%).

Characteristics	Categories	Study Sample	Resident Population
Gender	Women	52.1	50.0
	Men	47.9	50.0
Age	18–20	0.5	
	21–30	20.7	20.6
	31–40	24.9	19.6
	41–50	21.5	16.0
	51–60	14.4	14.8
	61–70	12.7	9.1
	70 and more	5.3	6.5
Education	middle school	12.3	34.3
	high school	15.7	21.2
	technical school	17.8	
	bachelor degree	38.8	37.5
	graduate degree	15.9	
Monthly household income	Less than 5000	12.3	
	5000–10,000	40.4	
	10,000–18,000	27.2	
	18,000–30,000	12.3	
	30,000 and more	7.8	

## References

[1] Nicklas T.A., O’Neil C.E., Fulgoni V.L. (2012). Diet Quality Is Inversely Related to Cardiovascular Risk Factors in Adults. J. Nutr. Dis..

[2] Bosire C., Stampfer M.J., Subar A.F., Park Y., Kirkpatrick S.I., Chiuve S.E., Hollenbeck A.R., Reedy J. (2013). Index-Based Dietary Patterns and the Risk of Prostate Cancer in the NIH-AARP Diet and Health Study. Am. J. Epidemiol..

[3] Popkin B.M. (2008). Will China’s Nutrition Transition Overwhelm Its Health Care System and Slow Economic Growth?. Health Aff..

[4] Wang Z.H. (2019). Dietary Structure Transition and Development of Nutrition Intervention Strategies in China. Acta Nutr. Sin..

[5] Chen P., Li F., Harmer P. (2019). Healthy China 2030: Moving from Blueprint to Action with a New Focus on Public Health. Lancet Public Health.

[6] Van Kleef E., Dagevos H. (2015). The Growing Role of Front-of-Pack Nutrition Profile Labeling: A Consumer Perspective on Key Issues and Controversies. Crit. Rev. Food Sci. Nutr..

[7] Trollman H., Jagtap S., Garcia-Garcia G., Harastani R., Colwill J., Trollman F. (2021). COVID-19 Demand-Induced Scarcity Effects on Nutrition and Environment: Investigating Mitigation Strategies for Eggs and Wheat Flour in the United Kingdom. Sustain. Prod. Consum..

[8] Zou P., Li Y., Liu Y. (2018). The Impact of Nutrition Label Regulation on Firm Marketing Action and Performance in China. J. Public Policy Mark..

[9] Ge K.Y., Jia J.B., Liu H. (2007). Food-Based Dietary Guidelines in China—Practices and Problems. Ann. Nutr. Metab..

[10] Lei L., Shimokawa S. (2020). Promoting Dietary Guidelines and Environmental Sustainability in China. China Econ. Rev..

[11] Zhang M., Che Y., Zhang Y. (2019). Systematic Review of Social Media Health Information Communication Behavior Research. Library.

[12] Visschers V.H.M., Hartmann C., Leins-Hess R., Dohle S., Siegrist M. (2013). A Consumer Segmentation of Nutrition Information Use and Its Relation to Food Consumption Behavior. Food Policy.

[13] Nie C., Zepeda L. (2011). Lifestyle Segmentation of US Food Shoppers to Examine Organic and Local Food Consumption. Appetite.

[14] Souiden N., Abdelaziz F.B., Fauconnier A. (2013). Nutrition Labelling: Employing Consumer Segmentation to Enhance Usefulness. J. Brand Manag..

[15] Asante-Addo C., Weible D. (2020). Profiling Consumers Based on Information Use and Trust in a Developing Economy. Int. J. Consum. Stud..

[16] Wongprawmas R., Mora C., Pellegrini N., Guiné R.P.F., Vittadini E. (2021). Food Choice Determinants and Perceptions of a Healthy Diet among Italian Consumers. Foods.

[17] Huang L., Bai L., Gong S. (2020). The Effects of Carrier, Benefit, and Perceived Trust in Information Channel on Functional Food Purchase Intention among Chinese Consume. Food Qual. Prefer..

[18] Zakowska-Biemans S., Pieniak Z., Gutkowska K., Wierzbicki J., Cieszynska K., Sajdakowska M., Kosicka-Gebska M. (2017). Beef Consumer Segment Profiles Based on Information Source Usage in Poland. Meat Sci..

[19] Bialkova S., Sasse L., Fenko A. (2016). The Role of Nutrition Labels and Advertising Claims in Altering Consumers’ Evaluation and Choice. Appetite.

[20] Grunert K.G., Wills J.M., Fernández-Celemín L. (2010). Nutrition Knowledge, and Use and Understanding of Nutrition Information on Food Labels among Consumers in the UK. Appetite.

[21] Sarmugam R., Worsley A. (2015). Dietary Behaviors, Impulsivity and Food Involvement: Identification of Three Consumer Segments. Nutrients.

[22] Thomas E. (2016). Food for Thought: Obstacles to Menu Labelling in Restaurants and Cafeterias. Public Health Nutr..

[23] Ducrot P., Julia C., Méjean C., Kesse-Guyot E., Touvier M., Fezeu L.K., Hercberg S., Péneau S. (2016). Impact of Different Front-of-Pack Nutrition Labels on Consumer Purchasing Intentions-A Randomized Controlled Trial. Am. J. Prev. Med..

[24] Michou M., Panagiotakos D.B., Lionis C., Costarelli V. (2019). Socioeconomic Inequalities in Relation to Health and Nutrition Literacy in Greece. Int. J. Food Sci. Nutr..

[25] Long N., Bach T., Huong T.N., Le H., Hoa D., Anh K.D., Cuong N., Carl L., Melvyn Z., Roger H. (2018). Socio-Economic Disparities in Attitude and Preference for Menu Labels among Vietnamese Restaurant Customers. Int. J. Environ. Res. Public Health.

[26] Huang Z.Y., Huang B.X., Huang J.Z. (2021). The Relationship between Nutrition Knowledge and Nutrition Facts Table Use in China: A Structural Equation Model. Int. J. Environ. Res. Public Health.

[27] Martinović I., Kim S.U., Katavić S.S. (2021). Study of Health Information Needs among Adolescents in Croatia Shows Distinct Gender Differences in Information Seeking Behavior. Health Inform. Libr. J..

[28] Agyemang-Duah W., Arthur-Holmes F., Peprah C., Adei D., Peprah P. (2020). Dynamics of Health Information-Seeking Behavior among Older Adults with Very low Incomes in Ghana: A Qualitative Study. BMC Public Health.

[29] Liu R., Hoefkens C., Verbeke W. (2015). Chinese Consumers’ Understanding and Use of a Food Nutrition Label and Their Determinants. Food Qual. Prefer..

[30] Siegrist M., Hartmann C., Lazzarini G.A. (2019). Healthy Choice Label Does Not Substantially Improve Consumers’ Ability to Select Healthier Cereals: Results of an Online Experiment. Br. J. Nutr..

[31] Smitha T.A., Valizadehb P., Linc B., Coats E. (2019). What Is Driving Increases in Dietary Quality in the United States?. Food Policy.

[32] Shangguan S., Afshin A., Shulkin M., Ma W., Marsden D., Smith J., Saheb-Kashaf M., Shi P., Micha R., Imamura F. (2019). A Meta-Analysis of Food Labeling Effects on Con-sumer Diet Behaviors and Industry Practices. Am. J. Prev. Med..

[33] Ollberding N.J., Wolf R.L., Contento I. (2010). Food Label Use and Its Relation to Dietary Intake among US Adults. J. Am. Diet. Assoc..

[34] Kiesel K., Villas-Boas S.B. (2013). Can Information Costs Affect Consumer Choice? Nutritional Labels in a Supermarket Experiment. Int. J. Ind. Organ..

[35] Kim M.G., Oh S.W., Han N.R., Song D., Um J.Y., Bae S.H., Kwon H., Lee C., Joh H., Hong S. (2014). Association between Nutrition Label Reading and Nu-trient Intake in Korean Adults: Korea National Health and Nutritional Examination Survey, 2007–2009 (KNHANES IV). Korean J. Fam. Med..

[36] Crockett R.A., King S.E., Marteau T.M., Prevost A.T., Bignardi G., Roberts N.W., Stubbs B., Hollands G.J., Jebb S.A. (2018). Nutritional Labelling for Healthier Food or Non-Alcoholic Drink Purchasing and Consumption. Cochrane Database of Systematic Reviews.

[37] Zhou Y., Wang E. (2011). Urban Consumers’ Attitudes towards the Safety of Milk Powder after the Melamine Scandal in 2008 and the Factors Influencing the Attitudes. China Agric. Econ. Rev..

[38] Mackison D., Wrieden W.L., Anderson A.S. (2010). Validity and Reliability Testing of a Short Questionnaire Developed to Assess Consumers’ Use, Understanding and Perception of Food Labels. Eur. J. Clin. Nutr..

[39] Palumboa R., Adinolfia P., Annarummaa C., Catinellob G., Tonellib M., Troianob E., Vezzosib S., Mannac R. (2019). Unravelling the Food Literacy Puzzle: Evidence from Italy. Food Policy.

[40] Vaitkeviciute R., Ball L.E., Harris N. (2015). The Relationship between Food Literacy and Dietary Intake in Adolescents: A Systematic Review. Public Health Nutr..

[41] Blaylock J., Smallwood D., Kassel K., Variyam J., Aldrich L. (1999). Economics, Food Choices, and Nutrition. Food Policy.

[42] Huang Z., Zeng D. (2021). Factors Affecting Salt Reduction Measure Adoption among Chinese Residents. Int. J. Environ. Res. Public Health.

[43] Ji W., Mao W.J., Dai W.B., Yu D. (2016). Thinking about China’ Promoting Food Education. Educ. Explor..

[44] Hou P., Wang L.E., .Liu X.J., Li Y., Xue L., Cheng C. (2018). Theory and Practice on Food Education Research at Home and Abroad. Resour. Sci..

[45] Guan L., Zhang Y., Jin S., Zhou L. (2021). Understanding the Low Use Rate of Food Nutrition Information in China. Int. Food Agribus. Manag. Rev..

[46] Bryła P. (2020). Who Reads Food Labels? Selected Predictors of Consumer Interest in Front-of-Package and Back-of-Package Labels during and after the Purchase. Nutrients.

[47] Li Y., Zhang X., Wang S. (2018). Health Information Quality in Social Media: An Analysis Based on the Features of Real and Fake Health Information. J. China Soc. Sci. Tech. Inform..

[48] Hou X., Huang C., Liu Y., Xie J. (2016). Research on Health Information Diffusion Model in We-media Based on Path Analysis. Inform. Sci..

[49] Bergeron S., Doyon M., Saulais L., Labrecque J.A. (2019). Using Insights from Behavioral Economics to Nudge Individuals towards Healthier Choices when Eating out: A Restaurant Experiment. Food Qual. Prefer..

[50] Kleef E., Seijdell K., Vingerhoeds M.H., de Wijk R.A., van Trijp H.C.M. (2018). The Effect of a Default-Based Nudge on the Choice of Whole Wheat Bread. Appetite.

[51] Stämpfli A.E., Stöckli S., Brunner T.A. (2017). A Nudge in a Healthier Direction: How Environmental Cues Help Restrained Eaters Pursue Their Weight-Control Goal. Appetite.

[52] Meengs J.S., Roe L.S., Rolls B.J. (2012). Vegetable Variety: An Effective Strategy to Increase Vegetable Intake in Adults. J. Acad. Nutr. Diet..

[53] Just D.R., Gabrielyan G. (2016). Why Behavioral Economics Matters to Global Food Policy. Glob. Food Secur..

[54] Middleton K.L., Jones J.L. (2000). Socially Desirable Response Sets: The Impact of Country Culture. Psychol. Mark..

